# What is your diagnosis?

**DOI:** 10.4274/jtgga.galenos.2019.2019.0043

**Published:** 2019-11-28

**Authors:** Nanthini Saravanan, Liji Sarah David, Reeta Vijayaselvi, Dipti Masih, Manisha Madhai Beck

**Affiliations:** 1 Department of Obstetrics and Gynaecology, Christian Medical College and Hospital, Vellore, South India; 2 Department of Pathology, Christian Medical College and Hospital, Vellore, South India

Mrs X, a 26-year-old primigravida woman presented for a morphology scan at 20 weeks of gestation. She had been married for 7 months and had conceived following one cycle of ovulation induction with clomiphene citrate. She was diagnosed as having gestational diabetes earlier in pregnancy and was started on a diabetic diet following which her sugars were controlled. She had an ultrasound scan at 6 weeks, which showed a single live intrauterine pregnancy corresponding to the gestational age. She had no history of intake of any teratogenic drugs. 

A morphology scan was suggestive of a monochorionic monoamniotic twin gestation. One of the twins was well formed and had normal anatomy and biometry corresponding to 20 weeks. Attached to the upper abdomen and thorax of the normal twin, was a significantly underdeveloped ‘co-twin’ that had only a trunk, both lower limbs, and rudimentary upper limbs (Figure 1).

The scan findings were discussed in details with the parents. Risks of surgical separation of conjoined twins, the chances of survival of the normal twin and the need for lower segment cesarean section as a mode of delivery in case the pregnancy was allowed to continue, were discussed. The parents opted for termination of pregnancy.

Autopsy findings revealed partial conjoined twins with a male autosite weighing 340 grams. The weight of the parasite could not be determined separately. The parasite co-twin was attached to the autosite at the level of the epigastrium.

The autosite showed a patent anus and no significant gross abnormality. The parasite had malformed limb buds, intestinal atresia, and a poorly developed spine (Figure 2a, b). Microscopic cut sections of umbilical vessels showed four arteries and a single umbilical vein.

## Answer

Epigastric heteropagus conjoined twins are very rare. They are also known as asymmetrical or parasitic conjoined twins, and are a rare complication of monozygotic twins (1). Their prevalence is 1 in 1-2 million births (2). The well-formed twin is known as an autosite, whereas its under developed counterpart is considered as a parasite because it is dependent on the former for its growth. The term ‘heteropagus’ was coined for the first time by Potter and Craig (3).

Asymmetrical conjoined twins are 20 times less common than symmetrical twins (4). Heteropagus twins are predominantly (78%) males, whereas symmetrical conjoined twins are mostly (70%) females. In our case also, both abortuses were males suggesting monozygotic twinning (5). Symmetrical conjoined twins share bowel and other organs, whereas asymmetrical twins do not share organs (6).

The pathophysiology of the heteropagus twinning has been explained in three theories.

The ‘fission’ theory suggests incomplete separation of the embryo (7), and the ‘fusion’ theory proposes coalition of two originally distinct parts (7). The third theory postulates that it occurs due to vascular compromise in utero, leading to death and partial resorption of one of the twins (7).

Most of the cases described in the literature were diagnosed postnatally (8). Few, however, were diagnosed prenatally like ours (9). The significance of the absence or presence of sharing of organs between the parasite and the autosite is that surgical separation is less complicated compared with symmetrical conjoined twins. There is still no consensus about the mainstay of therapy because the management of such cases is solely based upon case reports. When diagnosed early in pregnancy, termination may be a viable option especially in the developing world where surgery required for twin separation may not be widely accessible.

Surgical separation and closure of the incision may be attempted after babies are born. Wound breakdown is a dreaded complication following surgical separation (10). Long-term complications include hernia and teratoma at the incision site (11). Long-term follow-up of these babies following separation is very limited. One baby who was followed up to 52 months following surgical separation had normal growth and development (12).

## Figures and Tables

**Figure 1:**
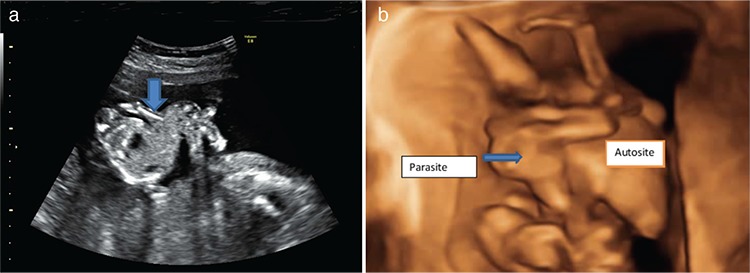
a) 2D ultrasound image showing transverse axial view of the upper abdomen of auto site with the stomach bubble. The arrow shows the origin of heteropagus with trunk, two lower limbs and malformed upper limbs, from the epigastric region of the autosite, b) 3D image of the epigastric heteropagus twin, parasitic twin arising from epigastric region of the autosite

**Figure 2:**
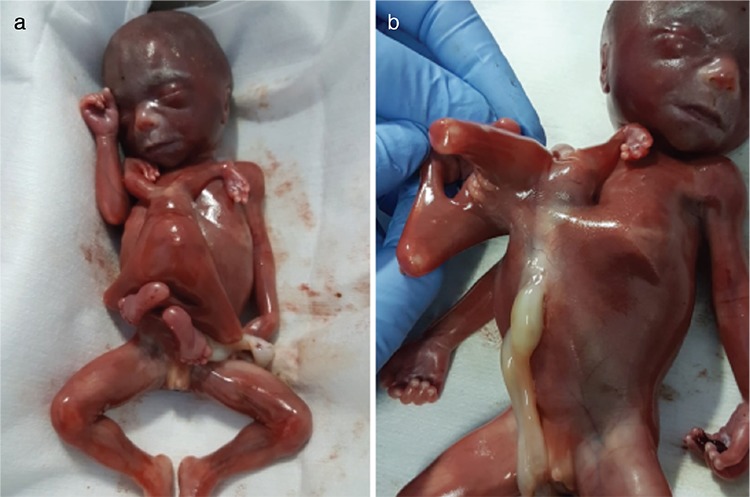
a) Gross specimen showing normal appearing male twin, arising from the epigastric region of which is the parasitic co-twin. b) Soft tissue pedicle connecting the parasite to the autosite and its relation to cord insertion on the autosite. Parasitic co-twin was also male with no skeletal muscle in the lower limbs
